# Nanotechnology in toothpaste: Fundamentals, trends, and safety

**DOI:** 10.1016/j.heliyon.2024.e24949

**Published:** 2024-01-20

**Authors:** Mehdi Abedi, Younes Ghasemi, Mohammad Mehdi Nemati

**Affiliations:** a Pharmaceutical Sciences Research Center, Shiraz University of Medical Sciences, Shiraz, Iran; b Department of Pharmaceutical Biotechnology, School of Pharmacy, Shiraz University of Medical Sciences, Shiraz, Iran

**Keywords:** Toothpaste, Nanoparticles, Hydroxyapatite, Hypersensitivity, Remineralization

## Abstract

Several studies have revealed that healthcare nanomaterials are widely used in numerous areas of dentistry, including prevention, diagnosis, treatment, and repair. Nanomaterials in dental cosmetics are utilized to enhance the efficacy of toothpaste and other mouthwashes. Nanoparticles are added to toothpastes for a variety of reasons, including dental decay prevention, remineralization, hypersensitivity reduction, brightening, and antibacterial qualities. In this review, the benefits and uses of many common nanomaterials found in toothpaste are outlined. Additionally, the capacity and clinical applications of nanoparticles as anti-bacterial, whitening, hypersensitivity, and remineralizing agents in the treatment of dental problems and periodontitis are discussed.

## Graphical abstract

**Figure:**
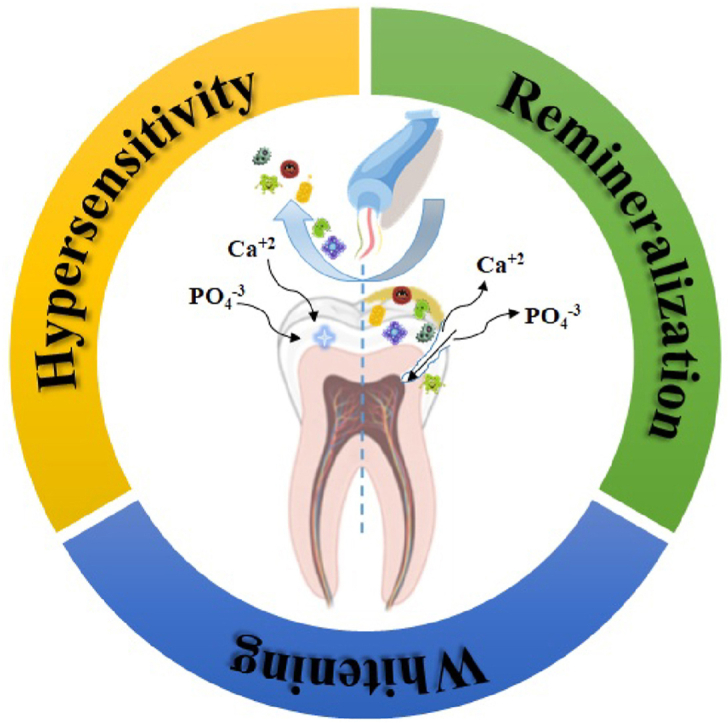

## 1 Introduction

Tooth decay is a worldwide illness that affects millions of people of all nationalities. Despite advances in early identification and treatment, dental caries remains the most common chronic bacterial-driven illness [1]. 2.3 billion adults are thought to have permanent tooth decay, and more than 530 million children are thought to have primary tooth decay, based on data collected from 195 countries by The Global Burden of Disease in 2017 [2]. Caries that are left untreated could spread into the dental core, result in dental lesions, cause excruciating pain, and eventually lead to dental decay. The activity of acids on the tooth surfaces is what causes tooth decay. When microorganisms in the tooth plaque break down the sugars in food or beverages, acids are created. Demineralization is the process of the enamel losing phosphate and calcium as due to the acid that is created [1]. It is critical to regulate oral bacteria biofilms on the surface of the tooth in order to prevent the development of cavities and dental problems [3].

**Fig. 1:**
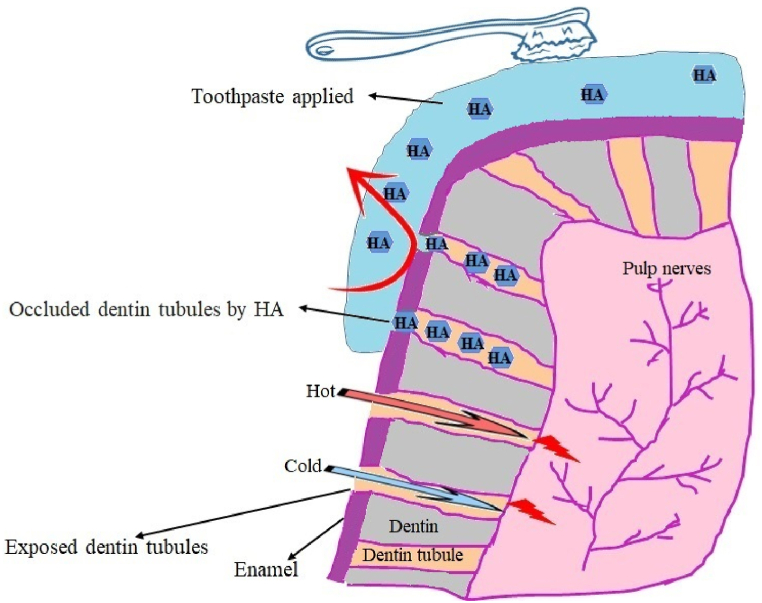
Nano-HAP toothpaste in reducing dental hypersensitivity.

In recent decades, there has been an upsurge in individuals with erosive tooth wear, which is clinically concerning [4]. Erosive tooth wear describes the degradation of the tooth matrix brought on by physical force, such as brushing, and reaction to oral cavity fluids [5]. These acids can come from foreign factors, such as citric acid-rich fruit drinks and soft beverages, or internal ones, such as gastric reflux, and can harm tooth compounds over time [6]. Compared to dentin, enamel has a different erosional mechanism. While hydroxyapatite (HAP) dissolution causes tooth enamel degradation, peritubular dentin dissolution causes it in dentin. It exposes collagen fibers of dentin to oral juice, resulting in hypersensitivity, demineralized dentin matrix, and loss of dental tissue [7]. Several factors, such as saliva chemistry and cavity-prevention ability, toothpaste composition, toothbrush types, and abrasiveness, may influence the interaction of acids and dental tissues, resulting in erosive tooth wear [5]. Several studies examined the impact of different toothpastes on enamel and dentin erosion. Numerous toothpastes include active ingredients, such as HAP nanoparticles (NPs), sodium fluoride (F), potassium nitrate, chitosan, and stannous salts, due to the beneficial role of these substances on damaged substrates [8,9]. These anti-erosive toothpastes, particularly those that include stannous ions, may lessen dentin hypersensitivity by producing a substance that could block tubular dentine, reducing the passage of tubular fluid brought on by environmental stimulation [10]. The Mechanism of NPs in threating hypersensitivity is graphically displayed in Fig. 1. Some toothpastes with anti-erosive claims may cause noticeable tooth abrasion [11]. There needs to be more agreement in the scientific literature on which toothpastes are best for patients with erosive tooth wear.

Nanotechnology might be critical in preventing dental disease through improved mouthwash and toothpaste [12]. Nanostructured materials, commonly known as "nanomaterials," are solid systems containing nanodomains embedded in a massive, dense matrix. This type of arrangement should be differentiated from "free" NPs, which are frequently generated as solid, scattered colloidal particles. Both are utilized in dental hygiene products that involve solid materials like toothpaste or liquid dispersion products like mouthwash, where component stability through manufacturing and aging is essential. Please keep in mind that, in our experience, most oral health care products have been reported to utilize just pure NPs since they require an intermediary solid or aqueous solution to avoid agglomeration, which limits the advantage of the nanoscale impact. The relatively new ability to synthesize pure "free" NPs enables the development of new performance systems featuring novel physicochemical and Bifunctional features, as well as nontoxicity [13].

As discussed in other application sectors dealing with nano dimensions, NP shape and size distribution may be essential elements for new dental hygiene applications [14]. However, since the size distribution is narrow and the shape is uniform, the expected nanometer-scale influence on NP characteristics is often believed to be significantly effective. The size distribution and forms of NPs are strongly related to their synthesis pathways and material composition. The overall stability of NPs, through production, sterilization, storage operations, and application in physiological environments, is also relevant in maintaining nanometric dispersion following the manufacturing and purification phases [15,16]. As a result, the final qualities of the final products (toothpaste and mouthwash) are determined mainly by the nanoparticles' dimensions. It is essential to investigate high-quality toothpaste in order to increase the performance of oral health care. Nanotechnology in nano toothpastes is a feasible solution. The pores of the enamel surface prisms cause bacterial accumulation in the porosities of the HAP, which are a significant part of the dentin enamel (about 70–80% dentin and 97% enamel) [3]. The nano-toothpaste would be beneficial in closing micropores, enhancing tooth appearance, and increasing the resistance of teeth against cavities [2,17]. In toothpaste, nanoparticles in the form of whitening agents such as TiO2, HAP, Charcoal, Carbon Nanotubes, and others are now used [[18], [19], [20]]. According to the American Academy of Cosmetic Dentistry, tooth-whitening products are one of the hottest cosmetic dentistry markets 2015 [21]. According to a study in China (Chengdu area), about 48.9 % of people have tooth discoloration, and about 52.6 % are unsatisfied with their tooth color [22]. Generally speaking, there are two teeth-whitening techniques: invasive and non-invasive. Invasive teeth whitening procedures involve crown restorations, which cause irreversible damage to teeth. In contrast, non-invasive whitening refers to teeth whitening using chemical methods [23].

The outer part of the tooth is composed of enamel, with most of the chemical composition consisting of HAP with a microstructure with a colored and semi-translucent appearance [24]. The outer layer of our teeth can change color from white to yellow due to tooth decay, diet, smoking, health conditions, trauma, and oral care status [25,26]. As you age, your teeth begin to decay, and the dentin layer is exposed. This layer of dentin is yellow. Even if you avoid pigmented foods and tobacco products, your enamel will be yellow with age [24,25]. However, some of our lifestyles and meals, such as smoking, coffee, tea, beverages, red wine, fruit juices, soy sauce, and so on, can cause teeth staining [27]. Different strategies, including bleaching, can resolve this type of discoloration, some specific dental cleaning by a dentist, laser whitening, and brushing with whitening toothpaste [28,29]. Whitening in this overview includes all means used to remove or prevent tooth discoloration, including photo etching, chemical bleaching, endobleaching, and laser whitening. Results regarding the effect of whitening agents on tooth roughness are mixed, but most researchers report an increase in enamel roughness after using whitening agents [30].

A commonly used teeth-whitening compound is H_2_O_2_ at a concentration of 25–35 %. H_2_0_2_ is a non-stable reagent that can cause several side effects, such as soft tissue irritation, tooth sensitivity, and the ability to inhibit pulpal enzymes. Therefore, there is a demand for an enhanced alternative and a low-cost option to teeth whitening [31].

Various ingredients must be employed by the manufacturer in order to formulate dental care products with multiple advantages. Advancements in nanotechnology have led to the development of products that function more efficiently and possess enhanced characteristics to benefit patients. When oral hygiene is more effective and more accessible to tolerate and, there are additional signals to remind patients, they are more likely to follow their healthcare provider's advice on preventing diseases. Preventing cavities is the most effective method to avoid dental issues. Brushing your teeth and keeping them clean is essential for a healthy mouth.

Moreover, the attractiveness of one's teeth plays a crucial role in boosting the inclination and requirement for using a tooth-whitening product that is safe. Damage to the dental enamel, the protective layer of the teeth, has been observed in numerous tooth-whitening methods and products available today. Due to the need for a comprehensive article about the role and importance of nanotechnology in toothpaste, which can evaluate various aspects and summarize nanomaterials, this point is mentioned in this article. The main subject of this paper is the ingredients comprising toothpaste, along with an analysis of recent studies regarding the utilization of nanomaterials in advanced toothpaste.

In order to gather information, we utilized the PubMed and Web of Science databases. We searched databases using specific keywords to find information. Keywords I used to be related to “nanoparticle”, “nano”, “nanotechnology”, “hydroxyapatite”, titanium dioxide, silver, gold, zinc oxide, chitosan, bioactive glass, silica, Sodium Triametaphosphate, Sodium Hexametaphosphate, Nanemulsions, Calcium Phosphate AND “toothpaste”, “dentifrice”, “oral care”, “mouthwash”, “anti-biofilm”, “anti-bacterial”, “anti-microbial”, “anti-inflammatory”, “remineralization”, “demineralization”, “hypersensitivity” and “whitening”. The research went on for a long time, starting in 1995 and ending in 2023. We reviewed the titles and abstracts of articles found in electronic databases as part of the selection process.

In our study, we discuss the incorporation of nanomaterials in oral care products, especially toothpaste, designed to combat bacteria, decrease sensitivity, remineralize, and enhance their whiteness. In this text, we are presented with information concerning the consequences of nanomaterials on living entities and their physical and chemical properties based on our current knowledge. However, the potential health impacts of being exposed to nanomaterials present in cosmetics are rarely mentioned or examined. The intention of this list is not to encompass all possibilities but rather to showcase the numerous ways nanotechnologies can be employed in dental hygiene products, ranging from toothpaste formulation to the prevention of tooth decay, combating bacteria, and improving tooth whiteness and sensitivity levels.

In recent years, the efficacy and quality of toothpaste and dental management have significantly increased by using nanotechnology in dental care equipment. In this review, we focused on and addressed innovative toothpaste and some recent studies on using nanomaterials, including phosphate-based, metallic nanomaterials, nano-emulsions, and chitosan NPs in toothpaste.

## 2 Toothpaste composation

Toothpaste is a semisolid gel composition applied to a toothbrush, promoting dental hygiene, maintaining teeth in good condition, and preventing discoloration and decay [32]. Currently, various types of toothpaste and densifiers for different purposes are available on the market. For each toothpaste, a different type of ingredient is added to the toothpaste formulation ingredients. However, some ingredients show multifunction capabilities for several purposes. Generally, toothpastes have these ingredients: Abrasive materials (about 50 % w/w formulation) that are used to smooth or polish surfaces (such as HAP, calcium carbonate, silica, and aluminum hydroxide); Anticaries agents such as sodium bicarbonate, xylitol, calcium and phosphate supplement, and F (1450 ppm in the form of NaF, Sn_2_F, and Na_2_PO_3_F) for tooth decay prevention and enhance tooth remineralization by accelerating the growth of fluorapatite crystals on the partially demineralized sub-surface crystals; Antibacterial agents (such as F, Ag, ZnCl2, triclosan, Sn^2+^, and herbal essential oils) to reduce plaque, gingivitis, and slightly reduces tooth decay; anti-plaque agents to assist plaque removal (such as sodium lauryl sulfate, triclosan, stannous-ions, zinc-ions, chlorhexidine); Whitening agents (such as papain, sodium bicarbonate, and abrasive material) to remove surface stains; solvents (about 20–40 % w/w, such as water and alcohol) for dissolving the ingredients and allowing them to be mixed as a smooth paste when you squeeze the tube and help the toothpaste from drying out; surfactant or foaming agents (such as sodium lauryl sulfate, and sodium lauryl sacrosinate) to create foam that caused to tooth cleaning, removing plaque and debris, humectants to prevent loss of water, harden of the paste, and provide a creamy texture (xylitol, propylene glycol, and polyethylene glycol, glycerol, and sorbitol); Antisensitivity agents (such as arginine, potassium nitrate, and strontium chloride) to relieve hypersensitivity; Anticalculus agents (such as zinc citrate, pyrophosphate, ureates, and sodium polyphosphate) reduce the calcification of dental plaque; Sweeteners (such as xylitol, glycerol, sodium saccharin, and sorbitol) to improve the taste of toothpastes and coloring agents for attractive appearance [33,34].

## 3 Nanomaterial

### 3.1 Phosphates-based material

#### 3.1.1 Hydroxyapatite nanoparticles

F has been considered the most effective caries-inhibiting agent for several decades; however, due to some side effect issues in children and lower public acceptance, another effective alternative is required [35]. Therefore, some researchers introduced HAP in toothpaste as a substitute for The F. In addition, HAP also acts as a source of calcium and phosphate ions, has a whitening effect, and reduces dental hypersensitization [36,37].

**Fig. 2:**
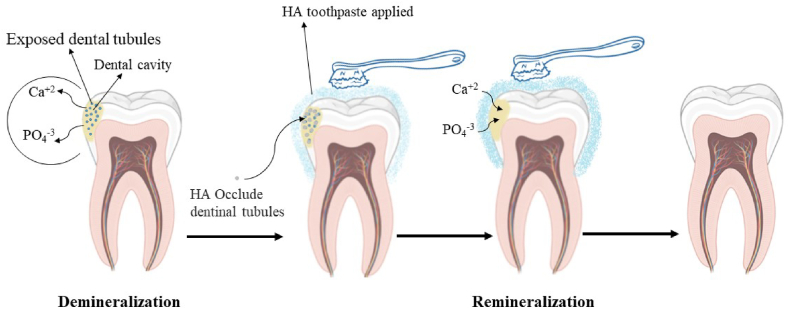
Demineralization and Remineralization process.

In general, tooth enamel is made up of HAP with a dense crystalline structure that extends from the dentin-enamel junction to the external enamel surface, which is surrounded by interenamel rods [38]. The enamel's capability to bear pressure resistance, protect against microorganism attacks, and reflect light [39]. Under a standard environment, the HAP minerals in the teeth shell and the oral liquids are dynamically balanced. Remineralization is the process of reintroducing phosphate and calcium ions from oral fluids into the tooth skeleton, as opposed to demineralization, which is the loss of phosphate and calcium ions as a result of a decrease in the pH of the oral fluid caused by bacterial activity. It has been determined that calcium and phosphate ion availability are two main factors restricting remineralization [38]. Demineralization and remineralization mechanisms induced by HAP are presented in Fig. 2. Calcium and phosphate make up the majority of the ingredients in HAP toothpaste. Micro- and Nano-HAP are similar to natural enamel HAP structures [36,40]. It has been demonstrated that HAP adheres to the enamel surface and penetrates the pore and crack to restore the surface integrity of the enamel. HAP particles have a more remarkable ability to penetrate the lesion than fluorides, which can only remineralize the surface [41,42]. It was established in Li's investigations that HAP NPs in the 20–40 nm range successfully accelerated the enamel-repairing process [43]. It has been established that HAP adheres to enamel surfaces, penetrates porous surfaces, and bends to enamel irregularities to restore surface integrity [44]. NASA first released HAP toothpaste, and then Japanese companies developed HAP toothpaste for tooth enamel repair, which was approved in Japan, Europe, and Canada in 1993, 2006, and 2015, respectively [39,45]. When seen under scanning electron microscopy (SEM), healthy enamel seems to have a surface that is smooth and unbroken. Demineralized enamel becomes porous after acidic treatments and has divots or other surface abnormalities [38]. It has been discovered that using toothpaste that contains HAP causes HAP crystals to adhere to the tooth surface, repair it, and fill in the pitted regions [38], whereas using F toothpaste did not fix any of the enamel surface imperfections. Studies demonstrated that teeth treated with HAP toothpaste had considerably greater calcium contents than untreated ones [46]. Surface hardness was also used as a reference to estimate the elemental composition of the teeth. Demineralized teeth are "fragile" and more sensitive to external forces [47].

HAP is among of the most exciting materials among tooth whiteners because its composition, crystalline phase, and microstructure closely resemble the apatite found in human tooth enamel [48]. Epple M assured that HAP particles have no adverse effects on human health and could be considered a biocompatible and non-toxic ingredient in oral care products when used in adequate doses [49]. In addition, Nano-HAP has shown positive results in people suffering from dentin hypersensitivity [50]. Niwa and colleagues report that HAP has a whitening effect on teeth even without brushing for the first time [51]. Their findings exhibited that the addition of HAP to the toothpaste did not have any significant effect on the polishing qualities. However, as the HAP content in the toothpaste increased, the whitening and bleaching power increased significantly. HAPs have been shown to adhere to tooth enamel and form a thin layer on the surface, causing light to reflect and giving teeth a shiny appearance [52]. However, many commercial mouthwashes use HAP as a whitening agent. Since long-term use results confirmed the teeth-whitening ability of Nano-HAP, consumers are choosing to use multi-functional toothpaste with various functions, including cleaning, protection, and whitening functions [53]. It is noticeable that HAP has a remineralization effect that increases the broad range of its capabilities, especially in children's dental care products [54]. Combining HAP-containing whitening toothpaste with commercially available toothpaste in a single product presents some complications due to considerations related to the interaction and stability properties of the various ingredients (e.g., HAP, F, and metaphosphate) [55].

**Fig. 3:**
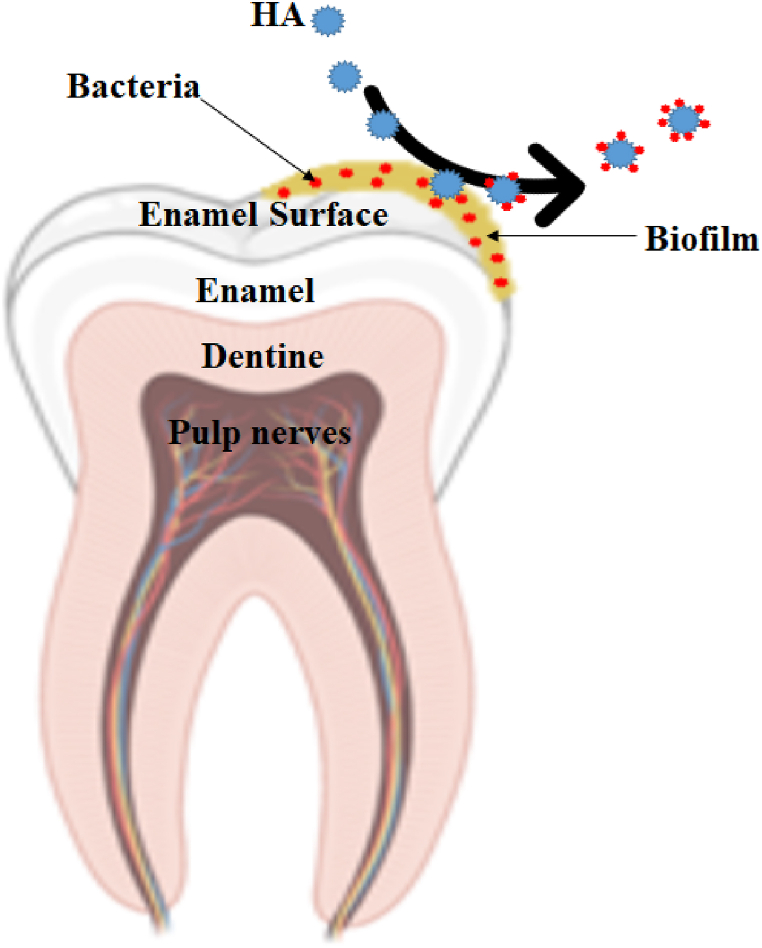
The mechanism of action of HAP on biofilm management. Through its interaction with bacterial adhesion, HAP can bind to microorganisms and expelling them from the oral cavity. Additionally, the pellicle receptors' ability to bind to bacterial receptors is blocked by HAP.

The findings confirmed that toothpaste products containing nano-whitening agents penetrate the microstructure of the teeth better, adhere firmly to the enamel, and cover a larger area of the tooth surface than products containing larger particles, and demonstrated improved protective and whitening effects [56,57]. Due to the larger surface area, the NPs have a larger contact surface with the enamel and form a more reactive particle with a more significant potential to form new strong bonds. In addition, literature data show that the concentration of HAPs plays an essential role in the whitening effect. Without the safety concerns, HAPs in commercial toothpaste can be increased up to 10 % to obtain an optimal whitening effect [53]. However, Kim et al. reported that prepared Nano-HAP toothpaste did not show any significant effect on teeth whitening in comparison to commercially available toothpaste containing micro-sized HAP, which showed similar whitening efficacy [58]. However, some research confirms the roles of HAP or Nano-HAP in tooth whitening and tooth sensitivity minimization [59]. In their in vivo studies, Sarembe and colleagues demonstrated that gels containing HAP have an external short-term whitening effect for a shorter time with no side effects or adverse effects on dentin and gingiva compared to other whitening agents in whitening toothpaste because most whitening toothpaste or toothpaste whitening agents contain abrasive or oxidizing agents (such as alumina, phosphates, hydrated silica, hydrogen peroxide, and perlite). The mechanism of the whitening effect of the prepared gel differs from other abrasive-based formulations because it is based on the adhesion of particles to the enamel surface [18]. Gomez and colleagues showed that treating teeth with HAP toothpaste before bleaching with 35 % hydrogen peroxide prevented the damaging effects of enamel [60]. Plus, Park et al. reported that toothpaste containing 15 % Nano-HAP, besides having a superior whitening effect over other whitening agents such as calcium carbonate and sodium metaphosphate, results in enamel remineralization and prevents bacterial colonization [61]. The mechanism of action of HAP on biofilm management is described graphically in Fig. 3. Table 1 shows the recent work on HAP and other nanoparticles used in toothpaste and their role in dental health care.

**Table 1:** Recent nanomaterial application in toothpaste.

NPs	Function Of NPs	Result
-Nano-HAP 7 % (pH = 6.94) -Zinc carbonate/Nano-HAP 24 wt% (pH = 7.34) -Aminefluoride 0.14 wt% (pH = 5.24) [40]	Remineralization	Toothpaste including Nano-HAP shows greater remineralization compared to toothpaste containing amine fluoride. The use of Nano-HAP (20 nm) leads to a remineralizing effect in the deeper parts of the lesion. The remineralization of Nano-HAP suspensions was enhanced by higher pH values.
-Nano-HAP/F -Nano-HAP [62]	Remineralization	Nano-HAP has remineralization potential. F addition has no synergistic effect on remineralization. The remineralization effect increases with the time.
Nano-HAP [63]	Remineralization	The presence of Nano-HAP in the formulation significantly increased enamel surface roughness compared to the F-treated group, but enamel roughness was significantly reduced in the Nano-HAP group.
-Nano-HAP (7%wt)/F [64]	Remineralization	Toothpastes containing HAP are more effective in remineralization and significantly increase microhardness
-Nano-HAP/F [65]	Remineralization	Although both anti-corrosion and anti-caries toothpastes reduced mineral loss to comparable levels, in vitro results revealed that F-free Nano-HAP toothpastes were ineffective at preventing cavity demineralization.
-Nano-HAP 1 wt%, 5 % Calcium sodium phosphosilicate −8% arginine, calcium carbonate, and 1450 ppm F [66]	Remineralization	The dramatic tubular occlusion feature of Nano-HAP toothpaste (approximately 98 %) that produces a resistant, protecting biomimetic layer on teeth may be the reason why it was more successful than the other ingredient in toothpaste.
Zinc carbonate substituted Nano-HAP -F, Mg, Sr-carbonate substituted Nano-HAP [67]	Remineralization	Zinc carbonate-substituted Nano-HAP toothpaste shows higher oral microbiota colonization and biofilm formation, as well as prevention of dental caries. F, Mg, and Sr carbonate-substituted Nano-HAP toothpaste reduces microbial colonization and biofilm formation due to F release.
Nano-HAP 10 wt% -Nano-HAP 10 wt%/950 ppm F −950 ppm F [68]	Remineralization	Nano-HAP 10 wt%-950 ppm F toothpaste significantly reduces lesion progress in comparison to other formulations and decreases the demagnetization rate of teeth.
Nano-HAP [69]	Remineralization	Toothpaste containing 5 % Nano-HAP effectively remineralized initial caries and inhibited demineralization of healthy enamel
Nano-HAP [70]	Remineralization	Nano-HAP showed enhanced remineralization capability in comparison to F varnish
Amorphous Calcium Phosphate, Nano-HAP and Calcium Sucrose Phosphate [71]	Remineralization	calcium sucrose phosphate shows a maximum increase in the enamel surface microhardness followed by Nano-HAP and Casein phospho peptide-amorphous calcium phosphate but Calcium Sucrose Phosphate in toothpaste strengthened the enamel more than Nano-HAP and CPP-ACP
Nano-HAP/NovaMin [72]	Remineralization	Both Nano-HAP and NovaMin (a synthetic mineral compound composed of calcium, sodium, phosphorus, and silica) were effective for remineralization of caries-like lesions of primary teeth and no significant difference was detected in their efficacy.
Nano-HAP [73]	Remineralization	Nano-HAP dentifrice caused remineralization comparable to a F dentifrice, and inhibited caries development
Nano-HAP/Tricalcium phosphate [74]	Remineralization	The study showed that Nano-HAP had the potential to remineralize artificial carious lesion better than TCP and F
Nano-HAP [75]	Remineralization	Nano-HAP and F had the potential to remineralize initial enamel lesions
Nano-HAP/F [76]	Remineralization	Nano-HAP paste were unable to reduce dental demineralization in vitro. But in present of F reduce the loss of enamel and dentin subsurface hardness
Nano-HAP [55]	Whitening	Nano-HAP toothpaste has a satisfying post brushing whitening effect and good resistance to mechanical forces. The whitening effect seemed to be concentration-dependent
Nano-HAP [77]	Whitening	toothpaste containing Nano-HAP provided a high degree of colour change in the short term and did not create significant surface roughness
Nano-HAP [78]	Hypersensitivity	combination of paste and liquid suspension with Nano-HAP for 14 days effectively reduced the hypersensitivity of the teeth with gingival recession
-Nano-HAP 15 wt% [79]	Hypersensitivity	The result of a randomized double-blind clinical study showed that Nano-HAP in F-free toothpastes significantly reduced sensitivity to air and touch (P 0.001).
-Nano-HAP 2 wt% gel [80]	Hypersensitivity	Toothpaste containing 2 % Nano-HAP significantly reduced tooth hypersensitivity
- KNO_3_, sodium Na_2_PO_3_F, and Nano-HAP plus antioxidants [81]	Hypersensitivity	KNO_3_ acts on neuroreceptors, while Na_2_PO_3_F and Nano-HAP affect renal tubule occlusion and show synergy in preventing hypersensitivity.
-Nano-HAP 10 wt% -Nano-HAP 15 wt% -Nano-HAP 10%wt/KNO_3_ -Calcium sodium phosphosilicate [82]	Hypersensitivity	Toothpaste containing Nano-HAP (15 %), Nano-HAP (10 %) wt/KNO3, and sodium calcium phosphate showed similar reductions in hypersensitivity when administered twice daily
-Nano-HAP 20 wt%/KNO_3_/NaF/F - Nano-HAP 10 wt%/KNO_3_/NaF/F -Arginine 8 wt%, Na_2_PO_3_F/F - NaF/F [83]	Hypersensitivity	After 3 months, a toothpaste containing Nano-HAP showed a similar reduction in hypersensitivity as other toothpastes without Nano-HAP.
Nano-HAP [84]	Hypersensitivity	Nano-HAP toothpaste shows a similar demineralization effect to F toothpaste, and can be used as an alternative anti-inflammatory agent for xerostomia patients and children.
Nano-HAP 7.5 wt%/F 100 ppm [85]	Hypersensitivity	Toothpaste with Nano-HAP and F reduces enamel weight loss and forms a fluorinated apatite layer.
Nano-HAP [86]	Hypersensitivity	After 7 days of treatment, Nano-HAP completes the tubular closure of the dentin and effectively reduces hypersensitivity, similar to bonding with G-Bond or Clear S3 Bond.
Nano-HAP [87]	Hypersensitivity	Toothpaste and mouthwash containing Nano-HAP and 8 % arginine showed a higher percentage of occluded dentinal tubules
Nano-HAP and 8 % Arginine [88]	Hypersensitivity	toothpaste containing arginine and provided a statistically significant reduction in
Nano-HAP [89]	Hypersensitivity	The HAP containing toothpaste was effective in reducing dentin hypersensitivity
Nano-HAP [90]	Hypersensitivity	Toothpaste containing 15 % Nano-HAP was found to be most effective in reduction of DH after a single application up to a period of 4 weeks followed by 8 % arginine
β-calcium glycerophosphate [91]	Remineralization	The addition of 0.25 % nano β-calcium glycerophosphate into toothpaste increased the bioavailability of Ca and P and promote enamel remineralization
mesoporous HAP [92]	Antimicrobial	thymol-loaded mesoporous HAP showed enhanced antibacterial activity and abrasive potential in comparison to thymol-loaded HAP due to sustained release behaviour
trimetaphosphate [93]	Demineralization	Adding about 3 % nano trimetaphosphate to a conventional toothpaste significantly decreased enamel demineralization
Nano-HAP [94]	Hypersensitivity	A meta-analysis compares different desensitizing toothpaste and result exhibited that Nano-HAP toothpastes may be the best desensitizing toothpastes for treatment of Hypersensitivity
nano casein phosphopeptides (nCPP)/nano amorphous calcium phosphate (nACP)/nano glycomacropeptide (nGMP) [95]	Antibacterial/remineralization	nCPP-nACP, and nGMP as a dental anticariogenic and remineralizing active agents
Nano monetite [96]	Remineralization	The toothpaste containing nano-monetite hydrosol exhibited the significant remineralization potential and acid-resistant compared with two commercial de-sensitive dentifrices comprising Nano-HAP and bioactive glass
F/Sn/chitosan [97]	Erosion prevention	The toothpaste containing F, stannous and chitosan shows promising results in reducing substance loss from erosion and abrasion. The combination of this toothpaste with the stannous-containing rinse showed even better prevention against erosion–abrasion.
nano-sized sodium trimetaphosphate and F [98]	Demineralization inhibition/Bleaching	Gels containing F/sodium trimetaphosphate do not interfere with the bleaching effect and reduce enamel demineralization
Ag and Au [99]	Antimicrobial	Silver in toothpaste has a greater antimicrobial effect than gold, but its effect is still inferior to that of a chemical antimicrobial agent
Ag NPs [100]	Antimicrobial	Combination of green synthesis of Ag NPs with commercial toothpaste enhanced antimicrobial activity of Toothpaste and prevent from biofilm formation, but this composition showed to be cytotoxic to the oral mucosa-representative. The toxicity is not a major problem because the toothpaste is not swallowed and only lingers in the mouth for a short amount of time.
Ag NPs/KF/silver diammine F (SDF) [101]	Antimicrobial/Remineralization	SDF caused change in collagen fibrils and intrafibrillar remineralization, SDF and Ag NPs/KF showed the greatest antibacterial effect
AgF [102]	Antimicrobial/Remineralization	The AgF toothpaste proved to be more efficient in eliminating bacteria and displayed a lower minimum inhibitory concentration (MIC) when compared to the conventional NaF toothpaste. Both toothpastes are equally effective in preventing tooth enamel demineralization.
AgF [103]	Antimicrobial/Remineralization	AgF proved to be more successful than NaF in preventing pH decrease and mutans adhesion on the enamel surface. Both NSF and NaF demonstrated effectiveness in tooth remineralization.
ZnONPs [104]	Antimicrobial	Addition of ZnO NPs in toothpaste ad concentration of 1 % shows significant antimicrobial activity against Streptococcus mutans
NaNbO3/ZnO [105]	Antimicrobial/whitening	NaNbO_3_/ZnO effectively degrade organic dyes that covered tooths and shows antibacterial capability against *Escherichia coli*
ZnONPs [106]	Antimicrobial/whitening	ZnO NP toothpaste effectively degrade oral biofilm formed on tooth
ZnONPs/F doped bioactive glass/TiO_2_	Abrasion	The restorative materials showed an insignificant difference in terms of micro-hardness before and after the treatment with all dentifrices
SiO_2_/Chitosan [107]	Increase retention time in oral mucosal surfaces	Addition of Surface medicated SiO2 NPs with some material such as chitosan, phenylboronic acid, and acryloyl groups caused to toothpaste retain in oral cavity for longer time after toothbrushing
Ca^2+^/PO_4_^3−^@Mesopore SiO_2_ [108]	Remineralization	Ca^2+^/PO_4_^3−^@MSNs occlude tubules and sustained release Ca^2+^/PO_4_^3−^
l-arginine-containing mesopore SiO_2_ [109]	Anti caries	l-arginine released in a sustained way from Mesopore SiO_2_ and diffused out from the dental adhesive, effectively contributing to the reduction of the bacteria.
Ag NPs [110]	Remineralization	Ag NPs inhibit 100 % microorganism growth, Ag NPs promoted remineralization tooth enamel with initial caries-like lesion and bactericidal activity
Ag NPs/calcium phosphate NPs [111]	Anti biofilm/Remineralization	Decreased biofilm viability and lactic acid production, useful in dental restorations to combat caries, Inhibiting growth of S. mutans
AgF [112]	Anti caries	AgF had a significant effect on the prevention demineralization of enamel and enamel caries
AgF [113]	Anti caries	AgF Arresting dentine caries, prevention from tooth discoloration
CuNPs [114]	Antimicrobial	Inhibiting growth of *S. mutans,* anticariogenic effects on root surfaces
Cu-chitosan [115]	Antimicrobial	Cu-chitosan exhibited superior capacity to prevent the S. mutans growth on human tooth surface in comparison to oral antimicrobial agents, such as chlorhexidine, and cetylpyridinium chloride
CuO-chitosan [116]	Antimicrobial	CuO-Chitosan NPs significantly enhanced in vitro antibacterial, antioxidant, cytotoxic activity and reduced secondary caries as compared to CuO NPs.
CuF2 [117]	Antimicrobial/anti caries	CuF2 decrease number of bacteria and a higher caries reduction in comparison to NaF and CuSO4
calcium phosphate NPs [118]	Remineralization	Inhibiting growth of bacteria and biofilm, increased biofilm calcium and phosphate content, promote remineralization
calcium phosphate NPs [119]	Remineralization	Calcium phosphate NPs showed strong antibacterial potency, inhibiting biofilm viability, promote remineralization
calcium phosphate/DMAHDM^a^ [120]	Remineralization	Root dentin hardness, strong antibacterial effects, Ca and P ion sustained release, inhibit root caries and protect tooth structures.
calcium phosphate/DMAHDM^a^ [121]	Remineralization	Strong antibacterial effects, reduced enamel demineralization and caries, increase hardness of enamel 4-fold
Calcium carbonate [122]	Remineralization	Release Ca in saliva and remineralization of enamel lesions
Carbonated Apatite [123]	Remineralization	Due to release in sustained manner Promoting remineralization, dentifrice containing 5 % n-CAPs showed the highest level of remineralization followed by 0 %, 15 % and 30 %, the dentifrice containing 5 % nano carbonated apatite and 25 % silica was the most effective in remineralizing early caries lesion.
CaF2-NPs [124]	Antimicrobial/Remineralization	CaF2-NPs substantially decrease the caries, about 90 % reduction in biofilm formation and exopolysaccharide production
CaF2-NPs [125]	remineralization	enhance the tooth remineralization, reduction of dentin permeability,
ZnO [126]	Anti caries	Improving integrity of the hybrid layer on caries-affected dentine
ZnO–Cu [127]	Anti caries	Significant antimicrobial activity, significantly inhibited Matrix metalloproteinase*,* improves the integrity of the hybrid layer on caries-affected dentin
Ag/ZnO [128]	Antimicrobial	Higher activity against to *S. mutans* compared with other antimicrobial agents
Ag/ZnO [129]	Antimicrobial	biofilm inhibition, increase compressive strength of enamel

a dimethylaminohexadecyl methacrylate.

#### 3.1.2 Calcium carbonate nanoparticles

Around 1850, calcium carbonate was first used in toothpaste formulas as an abrasive agent. The purpose of teeth cleaning with toothpaste at the time was to keep teeth shining and stain-free. F was introduced considerably later as just an anti-carry ingredient [130]. Fluoride's effectiveness as an anti-cavity agent has been demonstrated without a shadow of a doubt, and several experimental studies have shown the effectiveness of sodium F or sodium monofluorophosphate when added to a range of appropriate toothpaste compositions. Although plaque calcium and cavities have a well-documented inverse connection, toothpaste with a calcium carbonate base could also affect cavities by raising plaque calcium levels. The relationship between plaque calcium and F concentrations is also revealed. Hence, increased plaque F, which is related to less caries experience, may increase as a consequence of the use of toothpaste with calcium carbonate as an active ingredient. It has been established that calcium carbonate crystals are absorbed by plaque, which could affect caries by neutralizing the undesirable acids in the plaque while also releasing calcium [130].

Abrasive calcium carbonate is a component of several types of name-brand toothpaste. Abrasive toothpaste can wear down dental materials and enamel surfaces when used to brush teeth. Therefore, many studies have been done on surface roughness. Using toothpaste containing calcium carbonate has a significant effect on resin cement. In addition, the effects of micro- and nano-calcium carbonate toothpaste on the nanofiller composite resin were investigated. Surface roughness values were more significant in samples brushed with micro-calcium carbonate toothpaste than those treated with nano-calcium carbonate toothpaste [131]. The surface roughness was higher on nanofiller composite resin brushed with calcium carbonate toothpaste than on nano-calcium carbonate toothpaste. Due to variations in the size and roughness of the nano-calcium carbonate particles, different toothpaste brands have different stain-removing abilities. Calcium carbonate has also been proven to aid the process of remineralization. Currently, toothpaste formulations use calcium carbonate particles that range in size from 1 to 12 μm [132,133]. Toothpaste includes significant remineralization ingredients such as calcium carbonate and calcium phosphate. However, toothpaste with nano-calcium carbonate is already marketed, which could inhibit cavities since it has been demonstrated to remineralize initial caries lesions. Due to its small particle size, which ranges between tens and hundreds of nanometers, nano-calcium carbonate adheres well to the enamel surface. This size of the particles significantly increased the dissolving rate of calcium ions in nano-calcium carbonate, resulting in a rise in Ca^2+^ ion concentration and oral pH medium [122]. Rahardjo and colleagues investigate the effect of dental remineralization toothpastes containing calcium carbonate nano- and micro-sized particles. After a two-week treatment, each tested toothpaste was more successful in healing early cavities than the toothpaste without calcium carbonate. However, nano-calcium toothpaste shortens the time required for dental healing [134]. Anisja and colleagues demonstrated that 20 min of teeth brushing with calcium carbonate substantially increased tooth roughness, In contrast, typical toothpaste containing micro-calcium carbonate increased roughness more than nano-calcium carbonate toothpaste [135]. According to some studies, nano-calcium carbonate reduces the roughness of demineralized tooth enamel after brushing with nano-calcium carbonate toothpaste for a short period [136]. Brushing teeth with calcium carbonate toothpaste produced a rougher enamel surface than nano-calcium carbonate toothpaste and increased bacteria adhesion on the natural enamel and molded nanofill composite resin surfaces [131,133].

#### 3.1.3 Calcium phosphate nanoparticles

It is possible to stop demineralization from happening. If the microenvironment has a pH higher than seven and calcium and phosphate ions are present, remineralization can occur. On the tooth surfaces, these ions precipitate and create an amorphous mineral coating. However, in some cases, this layer might serve as a precursor to the emergence of crystalline structure [[137], [138], [139]]. Damaged dental mineral component prisms were shown to direct epitaxial development, thereby initiating a remineralization phase to repair demineralized tooth structure [140]. Despite differences in factors that affect their effectiveness, toothpaste is a viable and dependable method of delivering active ingredients to the tooth structure.

As a consequence, the addition of remineralizing agents to toothpaste has become a standard procedure [141,142]. Even though F is still widely used for preventing demineralizing reactions, calcium phosphate materials have been shown to accelerate remineralization processes [143,144]. As the most soluble CaP phase, CaP has been shown to release significantly more calcium and phosphate ions than other CaP phases, such as HAP. CaP is a metastable substance, though, and when it comes into contact with moisture or water, it instantly transforms into a variety of much more stable CaP phases, which is how it captures the transient stage of HAP synthesis [145]. Since some toothpastes contain calcium phosphate, oral calcium phosphate administration may cause a deposition of the tooth's mineral. After being swallowed, calcium phosphate particles travel to the stomach, dissolving completely at a pH of 1–2. As a result, their nanoparticle identity is completely lost, and they convert to HPO₄^2^⁻ and Ca^2+^. After passing through the stomach, it enters the intestine, which has a slightly alkaline pH. Calcium phosphate may precipitate under these conditions if its solubility is exceeded and nucleation is not inhibited by high concentrations of biomolecules [49,146]. The crystalline forms of beta-tricalcium phosphate (β-TCP), alpha-tricalcium phosphate (α-TCP), and amorphous calcium phosphate (ACP) are used in toothpaste and other oral care products. Hou and colleagues demonstrated that toothpaste containing calcium phosphate released calcium, which quickened enamel remineralization.

Furthermore, toothpaste containing a combination of β-TCP and ACP releases calcium faster than toothpaste entirely of ACP [147]. As mentioned, remineralizing tooth surfaces during mouthwash is greatly supported by increased free calcium and phosphate in the oral environment. From an oral care perspective, it would be advantageous to create a toothpaste suitable for daily use by adding various remineralizing agents that stop tooth decay without any side effects.

It has been demonstrated that nano-calcium phosphate shows an anti-sensitivity effect after whitening with a whitening agent. Generally, whitening agents such as hydrogen peroxide penetrate the dental pulp and result in inflammation and dental sensitivity. Currently, calcium phosphate is incorporated in the bleaching gel to reduce hypersensitivity to whitening toothpaste or other formulations. Calcium phosphate, similar to the HAP mechanism, blocks dental pulp and prevents hydrogen peroxide's side effects [148]. Three scientific studies demonstrated that applying calcium phosphate toothpaste decreases the sensitivity experienced from at-home tooth bleaching [59,149].

#### 3.1.4 Sodium triametaphosphate nanoparticles

Because of concerns about the efficacy of low-F toothpaste in caries management, researchers have been looking at ways to improve the anti-cavity impact of these products. Since sodium trimetaphosphate (TMP) plays a significant role in slowing down the dissolution of HAP, it has been used as an additive in toothpaste formulations [[150], [151], [152], [153]]. It is hypothesized that TMP adheres to the surface of tooth enamel, altering the affinity of the tooth surfaces for salivary proteins and reducing mineral exchange, HAP solubility, and demineralization of tooth enamel [154]. The addition of a small amount of TMP to a toothpaste containing F was found to have a more significant effect on enamel demineralization compared to its TMP-free counterpart. It has been demonstrated that the addition of TMP to toothpaste containing 500 ppm F has effects comparable to those of commercial toothpaste containing 1100 ppm F in terms of enamel demineralization, F, Ca, and insoluble extracellular polysaccharide in situ dental plaque [[154], [155], [156]]. These findings were supported by a randomized controlled trial in which children who used toothpaste with 500 ppm F and TMP exhibited significantly fewer decay lesions than those who used toothpaste with 1100 ppm F [157]. Notably, toothpaste containing TMP/F (250 ppm) showed similar anti-demineralization to traditional toothpaste, which only contained 1100 ppm F [158]. As nanoscale calcium phosphate has been demonstrated to help enhance enamel remineralization, it will also be essential to assess whether the addition of nanoscale TMP to usual toothpaste will have a synergistic effect on enamel remineralization compared to toothpaste containing microscale TMP and products that do not use TMP [159,160]. Emerenciano's studies showed that F/Nano-TMP toothpaste plus remineralization enhancement can substantially influence the structure of the biofilm formed on the enamel surface in comparison to fluoride-containing toothpaste [161]. In an in-situ approach, Souza et al. investigated the efficacy of a 250 ppm F toothpaste added with 0.05 % nanosized TMP to prevent enamel demineralization, changes in mineral composition (F, Ca, and P content), germs, and extracellular polysaccharide generation, and the results were compared with toothpaste containing 1100 ppm F. Ca, F, and P content were similar in both treated groups, but the F content of biofilm was higher in the group treated with 1100 PPM F than in the F/TMP group; however, the Ca and P content of biofilm were similar. Both treated groups showed similar surface hardness losses, but the F/TMP-treated group exhibited lower cross-sectional hardness [162]. The results showed that teeth treated with F/Nano-TMP had significantly higher atomic masses of Ca and P than those treatedwith toothpaste containing F/TMP or 1100F. Furthermore, the combination of 3 % Nano-TMP and F in toothpaste considerably decreased the extracellular carbohydrate matrix and increased the pH, P, and F content of the biofilm [163]. Comparison studies of different dental pulp capping formulations, including ZrO, TiO_2_, microTMP, and Nano-TMP, exhibited that formulations including Micro-TMP and Nano-TMP reduced setting times by about 50 %, and a reduction in TMP particle size increased their anti-cavity potential. Moreover, studies have shown that all investigated formulations showed antimicrobial activity against *S. mutan*s and *L. casei* [164]. Nanoscale compounds as an F alternative toothpaste formulation have gained popularity in the last decade due to their ability to reduce dental mineral loss. In this regard, the effect of nanoscale calcium phosphate toothpaste on enamel remineralization was also evaluated and exhibited considerable anti-cariogenic effects [165].

#### 3.1.5 Sodium hexametaphosphate nanoparticles

Since the last decade, several studies have been conducted to develop new toothpaste containing phosphate compounds to improve caries prevention. Another remineralizing substance, sodium hexametaphosphate (HMP), was launched in 2000 as an efficient anti-plaque component in toothpaste. It also has anti-inflammatory properties against periodontitis and can be used to alleviate tooth hypersensitivity [166]. This substance, polypyrophosphate, has various therapeutic and aesthetic effects. Sodium hexametaphosphate is a pyrophosphate derivative traditionally used to suppress discoloration and plaque. Because it comprises 10–12 repeating pyrophosphate subunits, it offers more coverage and adherence on the enamel surface than pyrophosphate. HMP prevents staining and removes residual stains due to its ability to bind dental minerals and dissociate plaque polypeptides containing stains [167]. Several scientific studies have demonstrated that HMP, sold as dentifrices and mouthwashes, has stain-removal and prevention properties [168,169]. Although HMP is commonly used as an anti-staining agent, it can also be used to prevent decay. HMP has a negative charge and is attracted by positive sites of enamel surfaces due to electrostatic attraction, causing remineralization of tooth enamel. It also contains three phosphate groups with free negative charge that act as functional groups to retain a positive bearing charge, such as calcium ions [168]. Saliva-soluble stains may seep into the freshly formed inorganic phase as the surface of the caries is mineralized during the remineralization phase in the oral cavity. The initial enamel caries' unsightly look, known as the "brown spot," is changed due to this occurrence. Some products nevertheless have disadvantages, including the possibility of staining.

Additionally, the beneficial attributes of HMP, combined with those of other remineralizing agents, may reduce the risk of surface discoloration and cavity development [170]. Remineralizing agents have synergistic effects when taken in combination. According to Camara DM studies, enamel remineralization by F and HMP combination toothpaste is equivalent to F-containing toothpaste [131]. In addition, when a toothpaste containing 1100 ppm F and 1 % HMP was combined, it had a more significant inhibitory impact on demineralization than a toothpaste that contained the same quantity of F [171]. According to Pfarrer and colleagues' reports, the F/HMP toothpaste exhibits improved anticavity performance compared to an F-containing toothpaste [172].

A small amount of sodium hexametaphosphate has a strong bond with the tooth enamel surface and, when combined with F, can reduce mineral loss [173]. Nanophosphate has also appeared as an advanced approach to optimizing the effect of F-containing toothpaste on the demineralization and remineralization processes. Dalpasquale research has shown that, due to the physicochemical properties of nanomaterials, conventional toothpaste containing Nano-HMP at a concentration of 0.5 reduces enamel demineralization and significantly increases the protective effect of the product compared to preparations containing Micro-HMP [174]. Sampaio et al. investigated the role of sodium hexametaphosphate micro- and NPs in the forming of saliva-derived biofilms in the presence and absence of F ions. Micro-HMP and Nano-HMP were shown to reduce colony-forming units and lactic acid generation in saliva drastically. Furthermore, Mico-HMP and Nano-HMP performed similarly and did not significantly differ (with or without F) [175].

Some researchers who combined sodium F (9 %) with the same amount of SHMP (9 %) found that toothpaste containing SHMP and F had a higher potential for remineralization than toothpaste including F alone. The combination of 0.5 % SHMP NPs with a traditional toothpaste with 1100-ppm F, on the other hand, accelerated the remineralization of artificial caries lesions and substantially altered the physicochemical characteristics of biofilm constituted with a higher amount of F and calcium, according to recent studies that examined the demineralizing effects of toothpaste enclosing Nano-HMP/F [161,173,176].

### 3.2 Metallic nanomaterials

#### 3.2.1 Titanium oxide nanoparticles

In Zhang's studies, a biocompatible polydopamine (PDA)-modified nano-TiO_2_ composite was prepared that was activated under blue light irradiation and showed a similar whitening effect to H_2_O_2_ but exhibited less enamel damage. In addition, this nanocomposite shows antibacterial function and ROS generation capability, which is a positive point for wound surface sterilization and biomedical application [177]. In the Istiqomah study, titanium dioxide nanotubes (TDN) were mixed with 3 % H_2_O_2_ and used as a tooth-whitening agent. The collected results showed that the prepared composite brightened the appearance of teeth by generating O_2_^−^ radicals [31]. Hamza and colleagues investigated TiO_2_ NPs as a teeth-whitening agent in two different concentrations (5 % and 10 % TiO_2_). The final result showed that the amount of TiO_2_ nanoparticle in the formulation had no effect on whitening, and the two formulations had similar effects [178]. To improve the efficiency of TiO_2_ as a bleach, Bulavinets and co-workers prepared Ag–TiO_2_ NPs. As Ag is deposited in the TiO_2_ NPs, the composite promotes surface plasmon resonance and enhanced electron-hole stripping activity, which increases the light-absorbing region in the visible region, resulting in improved bleaching activity of TiO_2_ NPs as a bleaching agent [179]. In a follow-up study by Bulavinet and colleagues, Kurzman prepared a photoactivated teeth-whitening gel containing different concentrations of TiO_2_/Ag and compared the results with different concentrations of hydrogen peroxide gel. Those who prepared gels containing TiO_2_/Ag improved the tooth-whitening effect compared to gels containing only TiO_2_, and the concentration of TiO_2_ in the gels showed no significant effect. Furthermore, gels including TiO_2_ or TiO_2_/Ag particles showed less cytotoxicity than those including hydrogen peroxide [180].

#### 3.2.2 Zinc oxide nanoparticles

Bacterial colonization of enamel is one of the critical risk factors for the occurrence of dental problems [181]. *S. mutans* is a bacterium thought to be the leading cause of tooth decay due to acid production that damages the external tooth tissues [182]. People with a high caries index widely colonize this microbe. As a result, eliminating these germs is critical for dental care. Bacterial reactions take place through a variety of mechanisms, such as metabolism exchange, accumulation, cell-cell interaction, and metabolism exchange [183,184]. These processes aid in the survival and proliferation of germs and the damage to the enamel and dentin of teeth produced by bacterial activity induced by tooth decay [185]. The improved antibacterial activity of NPs is due to the electrostatic interaction between negative charges on the interacting surfaces and positively charged NPs, which can inhibit or prevent the growth of more resistant strains. Some researchers report that zinc oxide NPs prevent the production of acid in Lactobacillus and *S. mutans* that are attached to tooth plaque [186]. It has also been shown to have antimicrobial properties against different pathogens. It is frequently employed as an antimicrobial ingredient in tooth hygiene products such as toothpaste due to its high surface-to-volume ratio [104]. Shahawi compares the antibacterial impact of toothpaste containing 0.5 % and 1 % ZnO to that of a control group. Their findings suggest that the presence of zinc oxide NPs improves antibacterial activity, and toothpaste with a higher concentration of ZnO has a superior impact [104]. Additionally, Prasad and colleagues demonstrated that using zinc oxide/F toothpaste twice daily dramatically reduced oral microbiota content compared to fluoride-only toothpaste [187]. Shaanxi Taihe Science & Technology Co. Ltd. has registered a patent for a product that is both safe and effective by using ZnO NPs as an alternative to F in toothpaste that eliminates dental plaque and gingivitis, as well as the effects of constriction and anti-bacteria, and encourages tissue repair [188].

#### 3.2.3 Silver and gold nanoparticles

The increased interest in precious metallic nanostructured materials has resulted in a rise in research and applications for such compounds. The literature contains data on the effectiveness of nanomaterials against gram-negative and gram-positive bacteria [189,190]. Among many metallic NPs, silver and gold NPs are two of the most famous metallic NPs [191]. As a result, some researchers focused on using silver and gold in toothpaste due to their superior antimicrobial activity [99]. Because of the excellent surface area-to-volume ratio, silver NPs in nanosized dimensions exhibit improved chemical reactivity [192]. Silver NPs diffuse through the bacteria's membrane, reducing membrane integrity and facilitating cell-external penetration [193]. Plus, during this process, silver NPs convert to silver ions and generate ROS that interfere with DNA replication, base pairing phenomena, and the protein production cycle in bacteria [193,194]. It causes cell death by disrupting the cell cycle and leaking via cell wall holes [195]. Because of their large surface free energy, NPs attach firmly to each other and other substances [195]. In vitro investigations have shown that silver NPs have potent antibacterial and antimicrobial activities. Aside from its antibacterial properties, nanosilver has also been demonstrated to have anti-inflammatory properties [196]. Toothpaste containing AgNP outperforms chitosan and F-containing toothpaste regarding antimicrobial activities against *S. mutans* [197]. In terms of reducing bacterial adhesion and pH reductions, nanosilver F toothpaste had a lower minimal inhibitory concentration and more statistically significant effects than NaF toothpaste. Compared to NaF toothpaste, nanosilver F mixtures are also efficient in avoiding bacterial adherence and pH changes. Nanosilver F and NaF were equally successful in preventing cavities by inhibiting enamel demineralization. Compared to NaF toothpaste, nanosilver F had a minor inhibitory concentration. Remineralization at the surface of the teeth did not occur due to Ag NPs treatment. At the same time, increased crystallinity may lead to increased stability of the apatite created at the tooth surfaces [198]. However, in some research, it is claimed that nanosilver can enhance cavity prevention and inhibit biofilm development [199]. In addition, Ag-loaded NPs are vital in the disinfection of dentinal tubules [200]. When used in oral care products, however, silver NPs in ions exhibit some toxicity [201]. Therefore, some manufacturers prefer to use silver NPs in a less toxic form, such as a colloidal form [202]. For better performance, Holladay assigned patent number US20130017236A1 in 2011 with a new silver nanoparticle formulation. He coated the silver nanoparticle with silver oxide and achieved superior antimicrobial activity compared toto other forms of colloidal silver formulation [203].

### 3.3 Nanemulsions

Nanoemulsions, intensively researched for various illnesses, are also a viable strategy for delivering medications to the oral cavity. Different ingredient agents can be formulated at the nanoscale to enhance their stability, solubility, permeability, loading, and release Using emulation technology. Researchers use emulsion formulations to increase effectiveness and reduce the adverse effects of some toothpaste ingredients. Meister E. et al. developed a novel hydrogen peroxide whitening emulsion to reduce the adverse effects of hydrogen peroxide. Their novel formulation confirmed bleaching action, but no abrasion loss was seen on enamel with the new formulation [204].

Lee and colleagues formulated a new toothpaste that continues to provide vitamin D for sublingual vitamin D delivery [205]. Vitamin D from oral ingestion is absorbed into the portal circulation from the intestines, which takes it to the liver first before entering the systemic circulation. The hepatic breakdown of vitamin D is a significant barrier that vitamin D molecules must overcome in order to reach the bloodstream. For sublingual vitamin D administration, Lee and colleagues created a novel toothpaste that contains vitamin D. When vitamin D is administered orally, the intestinal gut takes it into the portal circulation, which then transports it to the liver prior to it entering the bloodstream and degrades it into its metabolites. Vitamin D is deposited on the tongue and enters the systemic circulation via sublingual administration [206]. Their formulated vitamin D is in a water/olive oil emulsion-based toothpaste. Their findings revealed that intraoral vitamin administration is highly feasible.

Furthermore, the study discovered that their vitamin D toothpaste has similar properties to other commercially available types of toothpaste [205]. Some researchers studied administering vitamin B6 by including it in the toothpaste composition since it was considered an excellent fit, considering the significance of frequent administration of vitamin B6 without losing it, as most patients do. An emulsion-based toothpaste including vitamin B6, probiotics, and penetration enhancers was formulated and compared with commercially available toothpaste. The study found that the toothpaste produced was comparable to other commercial toothpaste and effectively controlled vitamin B6 administration [207].

### 3.4 Chitosan nanoparticles

Caries and dental problems are caused mainly by biofilm on the enamel surface and poor mouth and tooth conditions [181]. *S. mutan*s is regarded as one of the most common, highly bioavailable microorganisms. The increased activity and acidity of this bacterium produce polypeptides on the cell membrane that allow it to adhere to the teeth, which ultimately leads to the formation of biofilms and the formation of intracellular and extracellular carbohydrates that serve as attachment zones for many other germs to adapt to biofilms [182,208]. The biofilm that is formed reduces its susceptibility to the human immune system and other medications, resulting in a persistent bacterial infection [209]. Toothpaste is a commonly used hygienic tool for the management of dental plaque and the attainment of societally acceptable oral cosmesis. Regardless of formula variations, certain additives are found in a majority of toothpaste and serve specialized roles, such as water (moisturization), carbopol, carboxymethylcellulose, xanthan gum, and sodium alginate (agglutinant/thickener) [33,210], aromatizing agent (taste, mint, aroma, and sweetness) [131], wetting agent (hydrogen peroxide or activated charcoal) [20,211], antifoaming agent (silicon antifoam) [212], sodium bicarbonate and calcium phosphate-based agents (erosive action) [213,214], and an emulsifier, to aid in the removal of leftover food from the tooth structure [215]. Bioactive substances, such as antimicrobial agents like chlorhexidine, triclosan, F, and other natural compounds like herbal extracts, improve dental care [216,217]. As a result, it is crucial to select toothpaste with antibacterial properties [218,219].

Chitosan is a natural substance that interests the pharmaceutical and cosmetic industries. Studies have shown that chitosan has a wide range of antibacterial properties, affects cavity-prone microorganisms, adheres to bacteria in the oral cavity, limits their activity, and inhibits tooth demineralization and the development of bacterial plaque [220,221]. Chitosan microparticles may be used as F-delivery vehicles in dental plaque [222]. Despite some evidence to the contrary, chitosan appears to hold promise in the fight against erosion [223].

Arnaud et al. investigated the effect of chitosan on the demineralization process [215]. Their findings show that chitosan in tooth-care formulations inhibits tooth phosphate release and slows demineralization. Amount and contact time show a significant effect on the demineralization process and act as a defense against acid penetration [224]. Ahmed and colleagues examined the antimicrobial activity of three different toothpastes containing silver, chitosan, and F against *S. mutans*. All evaluated formulations have a robust antibacterial impact and efficiently prevent plaque development; however, nanosilver toothpaste has shown more antimicrobial activity than chitosan toothpaste [197].

Furthermore, chitosan toothpaste successfully reduces dental erosion [225]. In a study, Ganss and colleagues compare the erosion and abrasion effects of Na/F and F/Sn/Chitosan toothpaste. F/Sn/Chitosan toothpaste reduces organic tissue loss by 20–25 % compared to F-containing formulations. However, due to the presence of tin in the toothpaste, the sn/f formulation has a significant anti-erosive effect [226]. Francese and colleagues evaluated the protective effects of four different toothpastes, including TiF_4_, chitosan, TiF_4_/chitosan, and an erosion protection agent. Their findings showed that, despite chitosan, TIF_4_ toothpaste is the most effective agent for reducing erosive tooth wear, and the teeth of the chitosan toothpaste group are identical to those of the placebo group. TiF_4_ performs similarly to erosion protection toothpaste [225]. However, chitosan is utilized in specific toothpaste formulations to boost viscosity. Pavesi Pini and colleagues compared the protective and anti-abrasive effects of several toothpastes containing F/Sn, F/Sn/Chitosan (0.5 %, viscosity 50, 500, 1000, or 2000 mPas), and no F/Sn/Chitosan. Their findings revealed that the presence of chitosan in toothpaste as a viscose gel has a substantial influence on the performance of F/Sn toothpaste. Toothpaste containing chitosan and having a viscosity of 1000 mPas had the highest protective role, with increased surface preservation and decreased tin absorption by abrasives [223]. In another study, the combination of F/Sn/chitosan reduced tissue loss [226,227].

## 4 Safety and toxicity concern

Nanotechnology in cosmetics can be employed in various ways, producing nanomaterials with diverse characteristics and, consequently, different hazards and advantages. The importance and benefits of nanomaterials in cosmetics and medicine are undeniable. In recent years, many oral care products, such as toothpaste, mouthwash, dental fillings, and dental implants, have been used that have a unique property of nanomaterials [54,228,229]. However, the use of dental nanomaterials has not only provided considerable advancements in clinical care but has also raised increasing concerns about their biosafety [230,231]. Since nanomaterials are comparable in dimensions to DNA molecules, proteins, viruses, and biomolecules, some of their biological properties may be related to mechanisms of interaction between organisms and their conditions, which are currently unclear [232,233]. Therefore, many materials can exhibit significant cytotoxicity when reduced to the nanoscale. Calcium phosphate finds application in various cosmetic products, such as toothpaste. Sometimes, it can be used as a nanoparticle. Some worry about the impact that these NPs have on living organisms. Research has shown that calcium phosphate NPs do not have any severe harmful effects on their own. When calcium phosphate particles are swallowed, they go into the stomach and completely dissolve at a pH level of 1–2 [146]. The small calcium phosphate particles are unstable in highly acidic conditions [49]. However, when these NPs are taken into cells and degraded, they can cause an increase in the amount of calcium inside the cells. Apart from situations where excessive calcium phosphate is introduced, cells are capable of quickly eliminating calcium from their internal fluid. The observed harmful effect seen in certain cell culture studies is probably a result of the particles aggregating and sedimentation into the cell layer. Consequently, there is an elevated density of particles in a cell region, resulting in cell death. There is no reason to be concerned about taking calcium phosphate NPs by mouth because they quickly dissolve in the stomach, so there is no danger [49].

Over the past few years, there has been a significant surge in the usage of metal or metal oxide NPs such as CuO, ZnO, and Ag NPs, with a notable increase in applications. CuO, ZnO, and Ag NPs can hinder the growth and survival of bacteria, which can be detrimental to the health of people and animals. Additionally, they possess the capacity to combat viruses and cancer cells. Furthermore, they prevent the formation of biofilms, making them a potential substitute for traditional antibiotics [234]. However, due to the superior antibacterial and biofilm inhibition properties of using metallic or metal oxide NPs in oral care products, some concern about the safety of this material remained [235]. In toxicology, exposure route and dose are two critical factors. These NPs enter the bloodstream and migrate to different organs through the gastrointestinal tract after swallowing toothpaste foam. The absorption of NPs by the digestive system is influenced by various factors, such as the composition of the surface, the structure, the electrical charge, the dimensions, and the ability to bind with other substances [236]. The adverse side effects found in studies where Ag NPs were given by mouth were not very serious and only occurred when the dose was 125 mg/kg of body weight or higher [237].

Repetitive consumption of CuO, ZnO, and Ag NPs via oral administration can have detrimental consequences on your organs and result in inflammation within your body. CuO, ZnO, and Ag NPs accumulate in organs like the brain, lungs, liver, kidneys, and testes [238]. When the children's liver is exposed to NPs, it causes oxidative stress and apoptosis [239]. The administration of CuO and Ag NPs orally has caused disruptions in the functioning of the small intestine lining due to the impairment of microvilli. CuO, ZnO, and Ag NPs cause changes in the structure of liver tissue and make hepatocytes swell. Furthermore, they induce the accumulation of fluid around blood vessels, ultimately causing cellular death. It has been discovered that NPs can penetrate both the cell membrane and the mitochondria, which leads to cell damage and death when incubated with human gum cells [236,240,241].

The toxicity of NPs can vary depending on their shape, size, surface modification, morphology, and concentration. In conclusion, the NPs must be made less toxic to be safe for the environment and living organisms. By enhancing the surface modification, size adjustment, dissolution rate, and appropriate exposure method, the detrimental impacts of metal oxide NPs can be minimized. These preliminary results emphasize the need for in-depth research to enhance our understanding of how NPs induce toxicity [236,241]. Some toxicity side effects of oral administration of metallic and metal oxide NPs are presented in Table 2.

**Table 2:** Metallic and Metal oxide NPs related toxicity in oral administration.

CuO NPs
• Lung cancer [242]
• Oxidative stress and genotoxicity [243]
• Apoptosis and necrotic activity [244]
• Aggregation of mussel digestive gland [245]
**TiO**_**2**_**NPs**
• DNA-damaging potential [246]
• Genotoxicity [247]
• Accumulation of Ti in the liver, kidney, or spleen and strand breaks [246]
• Oxidative stress [248]
• Adverse cardiovascular effects [249]
• Intestinal tumor formation [250]
• Edema and fibrosis in the liver [250]
• Disturb glucose and lipid homeostasis [251]
• Hypoxemia [252]
• Lung cancer [253]
• Neurotoxicity [254]
Ag NPs
• Inflammation of the digestive tract [255]
• Imbalance of intestinal microbiota [256]
• Reduced the Thickness of the Intestinal Mucosal Layer [257]
• Decrease in the Abundance of Intestinal Microbiota [258]
• Apoptosis [259]
• Hepatotoxicity [260]
• Oxidative stress in brain [261]
• Mitochondrial ultrastructural changes [262]
• Central nervous system damage [263]
• Synaptic damage [264]
• Blood brain barrier disruption and brain edema formation [265]
ZnO NPs
• Accumulate in Heart, lung, liver, and kidney [266]
• Oxidative stress [267,268]
• Pulmonary inflammation and alveolar wall thickening [269]
• DNA damage and apoptosis [270]
• Liver apoptosis [271]
• Increase in the blood glucose level, degeneration of the cardiac muscle [272]
• Pancreatitis and anemia [273]
• Retinal atrophy, prostate inflammation [274]
• Toxic manifestations in the lymphatic system [275]

TiO_2_ was traditionally classified as physiologically nontoxic to both humans and animals. It was utilized as a control sample material in several toxicological investigations, although some toxic effects of TiO_2_ NPs on human health have lately been discovered. Similarly, some in vitro research has discovered that zinc oxide NPs, well-known for being nontoxic, are toxic to living cells [276,277]. Nanomaterials are not, in fact, intrinsically safe. Cellular, subcellular, and protein levels can affect biological activity at a various level. Some nanocomponents used in toothpaste or mouthwash can be swallowed, worn, or dissolved in the oral mucosa, enter the gastrointestinal tract, circulatory system, or central nervous system, and cause side effects [277]. Numerous studies conducted in recent years have shown that nanomaterials can accumulate in the kidneys, spleen, lungs, liver, and heart [278]. According to an investigation done in the Netherlands, it is possible that eating certain foods or using toothpaste could expose you to titanium dioxide NPs [279]. They calculated how much TiO_2_ NPs Dutch people consume daily through food, dietary supplements, and toothpaste. They were determined by using predetermined amounts of toothpaste and eating habits linked to diet data. Toothpaste, confectionery, and baked goods are the products that contribute most to the use of TiO_2_. Because children appear to be at risk of swallowing toothpaste, toothpaste accounts for 57 % of dietary TiO_2_ consumption in children.

Additionally, they computed the level of TiO_2_ NPs in human livers and compared it to the quantity found in test animals' livers, where side effects were observed. They advise more studies in order to acquire a better understanding of the potential impacts of prolonged exposure. Fadheela and colleagues tested different quantities of TiO_2_ NPs extracted from toothpaste on different human cell lines. The results showed TiO_2_ NPs were hazardous to the HepG2 liver cancer cell line. Furthermore, TiO_2_ NPs can cause human liver cancer at specific concentrations and exposure times [280].

Hsu and colleagues published a study in 2017 that found a link between TiO_2_ toothpaste and yellow nail syndrome [281]. Yellow nail syndrome is a rare condition characterized by lymphedema caused by the buildup of protein-rich fluid in the soft layers of tissue beneath the skin, swelling, trouble breathing, puffiness, yellowed or thickened nails, and other symptoms [282]. Hsu and colleagues collected their patient's nails and analyzed the amount of titanium in them. They discovered that the titanium content of their nails was more significant than average. This child's titanium intake was caused by her behaviour of swallowing children's toothpaste. With careful prevention of swallowing toothpaste, the patient's yellow nail discoloration improved, as did her lung problems.

In the mouth, a wide range of microorganisms, such as bacteria, yeast, protozoa, and viruses, can be present. The superiority of these microorganism microflora is due to their broad range of nutrients, suitable environment for colonization, and ability to survive on surfaces thanks to a biofilm [283]. A biofilm is a colony of bacteria in which microorganisms adhere to the surface and each other. However, habitant lifestyle, food diet, etc., may have a significant effect on the oral cavities’ live microorganisms and microflora content. As a consequence, the bacterial populations of the oral cavity differ widely over time; the content of the oral microbiota is not only variable but also very complex. The oral cavity is recognized as being associated with up to 1000 various bacterial species at 108–109 microbes per mL of saliva or mg of dental plaque, and research has proposed that only 50 % of the microorganisms growing at this site can be colonized [283]. Lactobacilli, Bifidobacterium, *L. reuteri*, *L. salivarius*, *L. paracasei*, etc. are safe and valuable microflora that generally live in the oral cavity [284]. These beneficial bacteria have a significant effect on food digestion (breaking sugar and proteins), neutralization activity, and colony of harmful bacteria (fighting with harmful bacteria such as *S. mutans* and maintaining a balance between beneficial and harmful bacteria), and protect teeth from cavities through saliva production [285,286]. These species effectively adhere to HAP NPs and are removed from the oral cavity, and increasing Ca^+2^ concentration, commonly found in toothpaste, enhances bacteria adhesion to HAP [287]. This non-selective nanoparticle antimicrobial activity can disturb the balance between beneficial and harmful bacteria or reduce the average population of bacteria in the oral cavity. By taking daily probiotic supplements after brushing your teeth every morning, you can reintroduce probiotic bacteria into your oral cavity. There are several ways to deliver beneficial bacteria into the mouth, including chewable tablets, mouthwash, and probiotic supplements containing specific probiotic strains. These bacteria then colonize your oral cavity and create biofilms that eliminate oral pathogens by competing with them [[288], [289], [290]].

In general, the oral cavity prevents substances from being absorbed through the mouth. It should be noted, however, that NPs are small enough to penetrate the oral cavity without difficulty [291]. As nanomaterials reach the buccal mucosa, they can alter the average physiological and biological balance in various. The buccal epithelium is directly affected by NPs, frequently associated with forming reactive oxygen species (ROS). For example, it has already been shown that TiO_2_ NPs can produce ROS and cause oxidative stress in lung cells that typically causes inflammation and apoptosis [292]. Second, free NPs might pass through the epithelium, reach the systemic circulation, and accumulate in the kidney, spleen, lung, and liver. As a result, several toxic consequences, such as liver cell damage and kidney damage, were found [293]. Whether NPs interact with the mucosae in the buccal mucosa or are ingested, however, is unclear. Investigating how NPs behave in saliva and determining how many of them are in the nano-scale range and capable of interacting with mouth mucosal membranes are thus of the highest relevance [291].

## 5 Future perspective

Nanotechnology is a field of science that focuses on studying and improving microscopic parts of materials like atoms or molecules [294]. Studying medicines, diagnosing illnesses, and enhancing our immune system are among the numerous domains heavily influenced by this. Through various applications of nanotechnology, a new field known as nanodentistry has been established in dentistry. Due to extensive research in biomaterials and nanotechnology, dental operations have seen significant improvements, offering a more comprehensive range of options. Unique features can be observed in nanomaterials, which more extensive materials do not possess [230]. When compared to traditional systems, nanodentistry offers numerous benefits. It can enhance whitening, remineralization, eradication of bacteria, biofilm removal, hypersensitivity capability, enhanced fillings, and improved cavity sealing. Despite its high cost, delicate placement requirements, potential toxicity, expensive development process, and restrictions set by international regulations, its use is necessary. Even with the challenges mentioned, scientists are currently making much effort to discover cheaper ways to create NPs. Despite the great potential of NPs, their use has some negative consequences. Their reduced stability, tendency to agglomerate, and the possibility of releasing metal ions or changing their composition by oxidation of their surface are associated with the problems associated with the use of nanoparticles. In the case of NPs, the degree of cytotoxicity may depend on the type of NPs, chemical purity, functionalization, preparation method, morphology, size, stability, and susceptibility to agglomeration. Studies have demonstrated that the toxicity of NPs can be reduced by different strategies, such as changing NPs' size, shape, and charge, as well as modification using ligands and coatings with other biocompatible reagents [295]. In particular, due to the effects of NPs on organisms after a period of use, it is still necessary to continuously study their biological compatibility. In order to ensure the safe use of these materials for medical applications, it is essential to understand how NPs behave in biological systems and their possible interactions with biological systems. Following the regulations and facilitating the development of NPs for oral care products, many new and different products have been researched and could be used by businesses. The testing of these materials revealed positive outcomes from their performance. Due to its superior advantage, capability, and chemical composition, it is deemed highly suitable as an active ingredient when formulating toothpaste for dental care [296]. Dentists express great satisfaction regarding utilizing these materials in numerous ways to create improved biomaterials. Enhanced utilization of nanotechnology and improvements in conventional dental care products could potentially enhance dental care [230].

## 6 Conclusion

Utilizing nanotechnology, scientists can create minuscule particles that hold significant potential in preventing tooth decay. Currently, toothpaste and mouth rinses contain different nanomaterials that can effectively prevent biofilm formation, enamel remineralization, dental hypertensives, and dentin discoloration. By applying nanotechnology to preventative dentistry, significant changes have been made. Several oral hygiene products incorporate these advancements. Dental hygiene products such as toothpaste and mouthwash have been infused with NPs with different properties, such as antihypersensitivity, antibacterial, and remineralizing capabilities. The versatile nature of nanomaterials enables their use in a variety of applications, and these applications are elicited by their promising outcomes and many, often undisclosed properties. However, NPs benefits including their surface characteristics, small size, diffusion in live cell, quantum properties, cellular uptake, mutation due to ROS and free radical production, are also their drawbacks. Because of the wide range of potential applications NPs, it is currently one of the most studied fields of science. Consequently, use of nanomaterial in toothpaste and mouthwash products must therefore be discussed and questioned due to regulatory concerns.

## Data availability statement

This is a review article, and Data sharing is not applicate to this article as no new data were created or analyzed in this study.

## CRediT authorship contribution statement

**Mehdi Abedi:** Writing – review & editing, Writing – original draft, Visualization, Validation, Resources, Methodology, Investigation, Conceptualization. **Younes Ghasemi:** Writing – review & editing, Supervision, Resources, Project administration, Data curation, Conceptualization. **Mohammad Mehdi Nemati:** Writing – original draft, Methodology, Investigation.

## Declaration of competing interest

The authors declare that they have no known competing financial interests or personal relationships that could have appeared to influence the work reported in this paper.
